# Expression and functional analysis of the hydrogen peroxide biosensors HyPer and HyPer2 in C2C12 myoblasts/myotubes and single skeletal muscle fibres

**DOI:** 10.1038/s41598-020-57821-1

**Published:** 2020-01-21

**Authors:** Escarlata Fernández-Puente, Manuel A. Sánchez-Martín, Jorge de Andrés, Lorena Rodríguez-Izquierdo, Lucía Méndez, Jesús Palomero

**Affiliations:** 1 Department of Physiology and Pharmacology, University of Salamanca, Salamanca, Spain; 2 Department of Medicine, University of Salamanca, Salamanca, Spain; 3 Transgenic Facility Unit, University of Salamanca, Salamanca, Spain; 4 Institute of Neurosciences of Castilla y León (INCyL), Salamanca, Spain; 5 Institute of Biomedical Research of Salamanca (IBSAL), Salamanca, Spain

**Keywords:** Ageing, Confocal microscopy, Fluorescent proteins, Multihormonal system disorders, Insulin signalling

## Abstract

Hydrogen peroxide (H_2_O_2_) is generated in cells and plays an important role as a signalling molecule. It has been reported that H_2_O_2_ is involved in physiological and pathological processes in skeletal muscle. However, H_2_O_2_ detection in cells with traditional techniques produces frequent artefacts. Currently, the HyPer biosensor detects intracellular H_2_O_2_ specifically in real time using fluorescence microscopy. The **aim** of this study was to develop and optimize approaches used to express the HyPer biosensor in different models of skeletal muscle cells, such as the C2C12 myoblast/myotube cell line and mature skeletal muscle fibres isolated from C57BL/6J mice, and to measure intracellular H_2_O_2_ in real time in these cells. The **results** show that the expression of the HyPer biosensor in skeletal muscle cells is possible. In addition, we demonstrate that HyPer is functional and that this biosensor detects changes and fluctuations in intracellular H_2_O_2_ in a reversible manner. The HyPer2 biosensor, which is a more advanced version of HyPer, presents improved properties in terms of sensitivity in detecting lower concentrations of H_2_O_2_ in skeletal muscle fibres. In **conclusion**, the expression of the HyPer biosensor in the different experimental models combined with fluorescence microscopy techniques is a powerful methodology to monitor and register intracellular H_2_O_2_ specifically in skeletal muscle. The **innovation** of the methodological approaches presented in this study may present new avenues for studying the role of H_2_O_2_ in skeletal muscle pathophysiology. Furthermore, the methodology may potentially be adapted to yield other specific biosensors for different reactive oxygen and nitrogen species or metabolites involved in cellular functions.

## Introduction

The historical view regarding the role of reactive oxygen species (ROS) in the cell has claimed that these molecules, ROS, are responsible for oxidative stress in the cell when they are present at high intracellular concentrations. When oxidative stress occurs, ROS react with lipids, proteins, carbohydrates and nucleic acids, which causes changes in the structure of these macromolecules that affect cellular functions and compromise cellular integrity, and this may in turn lead to pathophysiological states such as inflammation, cancer, neurodegenerative diseases and ageing, among others^1^. However, during the last twenty years, the understanding of the role of ROS in the cell has evolved, and now it is assumed in the field of redox biology that ROS play an important role in the regulation and modulation of different cellular functions, specifically when they are generated in the cell at lower intracellular concentrations compared to those that evoke oxidative stress^2,3^.

Hydrogen peroxide (H_2_O_2_) is one of the reactive oxygen species generated in cells together with other species, such as superoxide, hydroxyl radicals, nitric oxide and peroxynitrite^4,5^. It has been reported that hydrogen peroxide plays important roles in different cellular functions, such as energy metabolism, adaptive responses to different types of cellular stress and cellular growth^4,6,7^. The intracellular concentration of hydrogen peroxide determines the cellular function. Thus, at low intracellular concentrations (10^–8^ M), hydrogen peroxide may regulate cellular proliferation; at 10^–6^ M, H_2_O_2_ triggers cellular growth arrest; at 10^–4^ M, it evokes apoptosis^6,7^.

Skeletal muscle cells continuously generate reactive oxygen and nitrogen species (RONS), either at rest or during contractile activity^8,9^. Hydrogen peroxide is involved in physiological and pathological processes in skeletal muscle, such as contractile activity, passive stretching, wasting (sarcopenia), metabolic disorders (insulin resistance) and ageing, among others^5,10–15^. Furthermore, hydrogen peroxide is generated in different cellular compartments (i.e., the mitochondria, cytosol, endoplasmic reticulum, nucleus, and extracellular space), and the major sources for the generation of H_2_O_2_ are NADPH oxidases and the mitochondrial respiratory chain^16^. In addition, H_2_O_2_ is regulated by peroxiredoxins, glutathione peroxidases and catalase^7,17^. Moreover, H_2_O_2_ diffuses through cellular membranes, and its transport across membranes is facilitated by specialized membrane transport proteins known as aquaporins^18^. Thus, it appears that there is a flux of H_2_O_2_ through the different compartments of the cell.

To analyse in depth the role that H_2_O_2_ plays as a signalling molecule or a second messenger in cellular redox signalling and in oxidative stress, it is necessary to develop a reliable methodology to determine and quantify the concentration of hydrogen peroxide in different cellular compartments. Thus, the H_2_O_2_ flux in the cell may be assessed, since this may be the factor that modulates signalling pathways that regulate cellular functions. The main methodology used to determine intracellular hydrogen peroxide concentrations is based on a dichlorodihydrofluorescein (DCFH_2_) fluorescent probe. This has been a reliable method for determining hydrogen peroxide concentrations in different models, such as a skeletal muscle cell line model^19^, bundles of fibres from mouse diaphragm^20^ and single mature skeletal muscle fibres^21^. However, DCFH_2_ detects hydrogen peroxide, but it might detect other ROS at the same time and is not specific^22^. Other fluorescent probes have been used for H_2_O_2_ detection, such as dihydrorhodamine and boronates. These molecules may detect changes in the intracellular redox environment, but they are not specific for hydrogen peroxide and may be affected by other chemical interactions besides those caused by hydrogen peroxide generation^7,17,23,24^. Thus, these fluorescent probes may provide some information on cellular redox activity, but it might be misinterpreted. Currently, there are new genetically encoded probes available, such as HyPer and roGFP. These are new biosensors that provide important advantages for the detection of hydrogen peroxide in cells compared with the traditional florescent probes cited above^24^.

HyPer is the first genetically encoded biosensor used for the detection of H_2_O_2_ in cells^25^. This biosensor is an engineered and designed protein composed of two proteins; the circularly permuted yellow fluorescent protein (cpYFP) is inserted into the regulatory domain of the *Escherichia coli* protein OxyR, which is specifically sensitive for H_2_O_2_^25^. The main property of HyPer is that it reacts directly with H_2_O_2_ and forms a disulphide bridge, which leads to changes in the conformation of the protein that modify the spectrum of YFP. Thus, HyPer presents two excitation peaks at 420 and 500 nm, which correspond to the protonated (420 nm) and charged (500 nm) forms of the Tyr residue in the YFP chromophore, and one emission peak at 516 nm. These two forms can be visualized by fluorescence excitation at wavelengths of 420 and 500 nm by fluorescence microscopy^1^. When HyPer is exposed to H_2_O_2,_ the fluorescence emitted (at 520 nm) upon exposure to light at the excitation peak at 420 nm decreases in proportion to the increase in fluorescence emitted (at 520 nm) upon exposure to light at the excitation peak at 500 nm. This property makes it possible to carry out the ratiometric measurement of fluorescence, which is based on the calculation of the ratio of fluorescence (fluorescence emitted at 520 nm when HyPer is excited at 500 nm divided by the fluorescence emitted at 520 nm when HyPer is excited at 420 nm)^25,26^. An important advantage of ratiometric measurement is that this approach prevents artefacts associated with cell movement or differences in the level of HyPer expression. However, when cells do not change their shape or do not move, as in the case of adherent cells, which are the kind of cells used in this study, it is possible to monitor the fluorescence at a single wavelength^27^. This means using fluorescence excitation at 488 nm and measuring fluorescence emission at 512 nm. This is associated with the charged form of HyPer, which is the product of the reaction of H_2_O_2_ with HyPer. This is the approach we adopted in our study. Furthermore, due to the fluorescence properties of HyPer, this biosensor can be used as a detector of H_2_O_2_, and in combination with fluorescence microscopy imaging analysis, it is possible to detect changes in the intracellular concentration of H_2_O_2_ and to quantify in some way the intracellular flux of H_2_O_2_. The high reactivity and selectivity of HyPer towards H_2_O_2_, the possibility of ratiometric detection, the reversible oxidation of HyPer and its ability to target different tissues and subcellular compartments make HyPer a promising biosensor to study the flux of H_2_O_2_ analytically in skeletal muscle cells.

The objective of this study was to develop approaches to express the biosensor HyPer in different models of skeletal muscle cells where, in combination with fluorescence microscopy imaging techniques, it might be possible to measure intracellular changes in the concentration of H_2_O_2_ in skeletal muscle cells in real time. Three models of skeletal muscle cells that have been used in the field of skeletal muscle biology were explored: the mouse myoblast cell line C2C12, C2C12 myotubes, which are differentiated from C2C12 myoblasts, and single mature skeletal muscle fibres isolated from the *flexor digitorum brevis* muscle in mice.

## Results

### HyPer expression in C2C12 myoblasts

**Figure 1:**
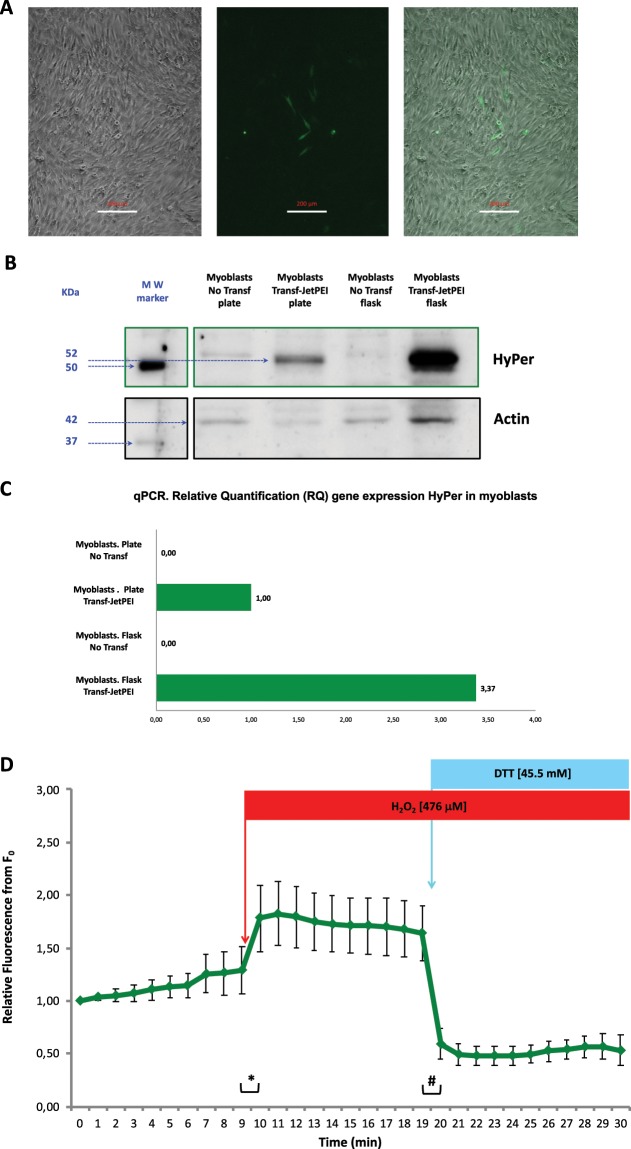
C2C12 myoblasts after transfection with the pHyPer-cyto vector using the transfection reagent JetPEI. (**A**) Microscopy images: Bright-field image (left), fluorescence image (centre) and merged bright-field and fluorescence image (right). Scale bar 200 μm. (**B**) Cropped immunoblotting images of HyPer protein expression. Myoblasts were grown either on plastic culture plates or in plastic culture flasks. The MW marker represents the lane where the molecular weight protein reference markers were resolved in the immunoblot. HyPer and actin proteins were detected. Full-length immunoblot image in Supplementary Information. (**C**) Gene expression of the HyPer DNA coding sequence. Relative quantification (RQ) of the HyPer mRNA transcript by using qPCR; the housekeeping gene used for normalization was the mouse beta-actin gene. Myoblasts that were transfected or not transfected (control) were cultured either on plates or in plastic culture flasks. The reference used for the relative quantification of HyPer gene expression were myoblasts that were transfected and cultured on plates. (**D**) HyPer fluorescence emission from individual myoblasts that expressed HyPer. Fluorescence was monitored every minute over a 30-minute time course. The mean values of relative fluorescence are presented ± s.e.m., n = 6 independent myoblasts. Myoblasts were exposed to H_2_O_2_ at a 476 μM final concentration in medium at the 9–10 min time point and were exposed to DTT at a 45.5 mM final concentration in medium at the 19–20 min time point. * and ^**♯**^ statistically significant (p < 0.05) according to Student’s *t*-test.

The expression of the biosensor HyPer in C2C12 myoblasts was achieved by transfection of the pHyPer-cyto vector, a plasmid into which the coding DNA sequence of the biosensor HyPer is incorporated, into C2C12 cells. We performed a chemical transfection protocol based on the reagent JetPEI (Polyplus Transfection) at a ratio of 6 μg DNA: 12 μl JetPEI per 35 mm dish well with C2C12 myoblasts in culture at 80% confluence. HyPer expression in these cells was assessed 24–48 hour after transfection by fluorescence microscopy. Although the efficiency of transfection was apparently low, we found several myoblasts that expressed a fluorescence protein that might be the biosensor HyPer (Fig. 1A).

To assess and identify the fluorescence protein expressed in the C2C12 myoblasts, we applied immunoblotting techniques (Western blotting). C2C12 myoblasts were transfected with pHyPer-cyto vector and JetPEI, and a few cells (1–2% of counted cells in some fields) expressed a fluorescent protein. Immunoblotting analysis was carried out, and the results revealed that myoblasts that had been cultured either in plates or flasks and transfected with JetPEI and pHyPer-cyto vector contained a 52 KDa protein that was recognized by a specific antibody, anti-HyPer (Evrogen, AB121) (Fig. 1B). This indicated the presence of the HyPer protein.

Additionally, to determine whether the 52 KDa fluorescent protein that we observed with fluorescence microscopy and immunoblotting was the biosensor HyPer, we performed gene expression analysis by applying the methodology of quantitative real-time PCR (qPCR). Specific primers that matched the DNA coding sequence of HyPer were designed and used for qPCR in combination with specific primers that matched the endogenous mouse gene beta-Actin sequence (mActb). The results of the relative quantification (RQ), which used HyPer gene expression in myoblasts that had been cultured in plates and transfected with JetPEI and pHyPer-cyto as a reference (1.00), revealed that the expression of the HyPer encoding DNA sequence, which was inserted in the pHyPer-cyto vector, was present in C2C12 myoblasts that had been transfected with JetPEI and pHyPer-cyto. As expected, the negative controls, which were C2C12 myoblast cultures either in plates or flasks that had not been transfected, did not show HyPer gene expression (Fig. 1C).

The most relevant aspect of our study, apart from achieving the expression of the biosensor HyPer in skeletal muscle cells, was obtaining a functional biosensor. Thus, in the case of HyPer, the protein should be efficient in emitting fluorescence when it reacts with hydrogen peroxide so that it can consequently detect changes in intracellular hydrogen peroxide concentration. Figure 1D presents the results of a group of independent fluorescence microscopy live-cell experiments in myoblasts (n = 6) that expressed HyPer. The fluorescence in live myoblasts in culture was monitored during a 30-min period by acquiring digital images every minute. The analysis of the digital images from individual myoblast ROIs revealed the quantitative fluorescence values of the myoblasts at every time point. Thus, it is possible to assume that this is a method that can be used to quantify real-time fluorescence emitted by C2C12 myoblasts that express HyPer. The functionality of HyPer in these experiments was assessed by the addition of hydrogen peroxide to the medium at the 9–10 min time point and the addition of a reducing reagent, dithiothreitol (DTT), to the medium at the 19–20 min time point. The results showed that after the addition of hydrogen peroxide to the medium (final [H_2_O_2_] 476 μM), HyPer fluorescence in myoblasts increased dramatically and with statistical significance. Following the addition of dithiothreitol to the medium (final [DTT] 45.5 mM), a dramatic and statistically significant decrease in HyPer fluorescence in myoblasts was observed. In fact, HyPer fluorescence decreased below the basal values that were observed at the beginning of the time-lapse experiment (Fig. 1D).

### HyPer expression in C2C12 myotubes

**Figure 2:**
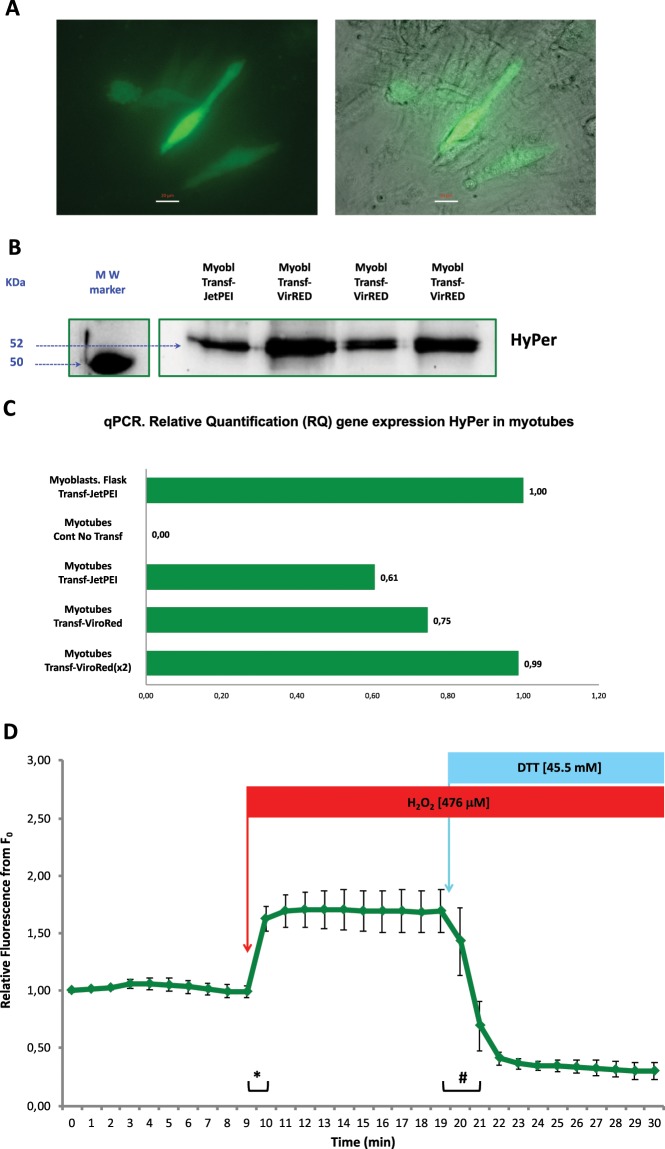
C2C12 myotubes after transfection with the pHyPer-cyto vector using the transfection reagent Viromer RED. (**A**) Fluorescence microscopy image (left) and merged bright-field and fluorescence microscopy image (right). Scale bar 20 μm. (**B**) Cropped immunoblotting images of HyPer protein expression in C2C12 myoblasts that were previously transfected with pHyPer-cyto vector using the transfection reagent JetPEI or Viromer RED. The MW marker represents the lane where the molecular weight protein reference markers were resolved in the immunoblot. Full-length immunoblot image in Supplementary Information. (**C**) Gene expression of the HyPer DNA coding sequence. Relative quantification (RQ) via qPCR of the HyPer mRNA transcript; the housekeeping gene used for normalization was the mouse beta-actin gene. C2C12 myotubes that were not transfected (control) or transfected with pHyPer-cyto vector using the transfection reagent JetPEI or Viromer RED were cultured on plastic culture plates. The reference used for the relative quantification of gene expression were myoblasts transfected with pHyPer-cyto vector using the transfection reagent JetPEI and cultured in flasks. x2 represents myotubes that were transfected with the pHyPer-cyto vector using twice the amount of Viromer RED normally used in the transfection procedure. (**D**) HyPer fluorescence emission from individual myotubes that expressed HyPer. Fluorescence was monitored every minute over a 30-minute time course. The mean values of relative fluorescence are presented ± s.e.m., n = 5 independent myotubes. Myotubes were exposed to H_2_O_2_ at a 476 μM final concentration in medium at the 9–10 min time point and were exposed to DTT at a 45.5 mM final concentration in medium at the 19–20 min time point. * and ^♯^ statistically significant (p < 0.05) according to Student’s *t*-test.

The second objective of this study was to achieve the expression of the biosensor HyPer in myotubes. Myotubes are differentiated skeletal muscle cells that are formed from the fusion of C2C12 myoblasts, which generate multinucleated cells with a tubular morphology^28^. During the embryonic development of skeletal muscle, the myotube is an intermediate cellular state between the myoblast and the mature skeletal muscle fibre^29^. First, we transfected the pHyPer-cyto vector by using JetPEI into myotube cultures using the same protocol that we applied to myoblasts (see above). However, the results of transfection were limited in terms of the effectiveness of HyPer expression, but we found positively transfected myotubes that expressed HyPer 14 days after transfection; in addition, HyPer was functional and responded to the addition of hydrogen peroxide and DTT, which resulted in the augmentation and the decrease of HyPer fluorescence, respectively. To improve the effectiveness of transfection and HyPer expression, we assayed a different chemical reagent for transfection, Viromer RED (Lypocalyx). According to the manufacturer, Viromer RED is a chemical polymeric transfection reagent that takes advantage of a viral membrane fusion mechanism that facilitates endocytosis, transport across the membrane and the delivery of genetic material into the cytosol of the cell. Figure 2A shows myotube cultures transfected with pHyPer-cyto vector and Viromer RED (transfection was performed in myoblasts, which differentiated into myotubes 24 hours after transfection). Images show myotubes that expressed HyPer six days after transfection (Fig. 2A).

Figure 2B presents an immunoblotting image that reveals the expression of HyPer in 3 samples of protein homogenates from myoblast cultures that were transfected with pHyPer-cyto vector and Viromer RED and one sample of protein homogenate from myoblasts transfected with pHyPer-cyto vector and JetPEI, which was the positive control reference for the expression of the biosensor HyPer in these cells.

The gene expression analysis of HyPer in myotubes is presented in Fig. 2C. A sample of myoblasts transfected with the pHyPer-cyto vector and JetPEI was the reference (1.00) for the relative quantification (RQ) of HyPer gene expression. As expected, the experimental negative control (untransfected myotubes) did not show any signal of cDNA amplification, indicating that HyPer gene expression was absent. A sample of myotubes transfected with the pHyPer-cyto vector and JetPEI showed lower HyPer expression (0.61) than the reference (1.00). Myotubes transfected with the pHyPer-cyto vector and Viromer RED showed HyPer expression levels of 0.75 and of 0.98, respectively, when twice the amount of transfection reagent (Viromer RED) was used in the transfection protocol.

To assess HyPer biosensor function (via the detection of changes in the intracellular hydrogen peroxide concentration) in myotubes, we conducted live cell fluorescence imaging experiments under the microscope. Figure 2D shows the fluorescence imaging analysis from these experiments. Independent myotube cultures were transfected with pHyPer-cyto vector and Viromer RED (using twice the amount used in the standard protocol), and some myotubes were located and assessed for HyPer expression via live cell fluorescence imaging experiments. C2C12 myotubes that expressed HyPer were imaged, and the fluorescence emitted by these cells was monitored in real time by digital imaging recording. The addition of hydrogen peroxide to the medium (final [H_2_O_2_] 476 μM) immediately after the 9 min time point of the time-lapse experiment evoked a rapid and significant intracellular fluorescence increase in myotubes that expressed HyPer, and the addition of dithiothreitol (final [DTT] 45.5 mM) just after the 19 min time point of the time-lapse experiment evoked a significant decrease in intracellular fluorescence. Furthermore, the fluorescence level of the myotubes after DTT addition was lower than the basal level of fluorescence at the beginning of the time-lapse experiment (Fig. 2D).

### HyPer expression in single muscle fibres isolated from the FDB mouse muscle

Previously presented results made it possible to achieve the main objective of this study, which was the development and optimization of a plausible method to obtain the optimal expression of the biosensor HyPer in single isolated skeletal muscle fibres from the FDB mouse muscle. Initially, we attempted the transfection of the pHyPer-cyto vector with either JetPEI or Viromer RED into cultures of single muscle fibres previously isolated from FDB muscle. We used the same protocols that utilized chemical transfection with either JetPEI or Viromer RED, which had been effective for the expression of HyPer in myoblasts and myotubes, as described in the previous results above. In addition, we introduced some modifications to the protocols to adapt them for use in isolated muscle fibres. However, the results were unsatisfactory in terms of the expression of the biosensor HyPer in single isolated muscle fibres. This led us to explore a different methodological approach to obtain the proper expression of the biosensor HyPer in single skeletal muscle fibres. Previous studies described a method to transfect DNA into skeletal muscle using *in vivo* electroporation^30^. Based on this methodology, we incorporated some adaptations for the single skeletal muscle fibre model and designed a protocol to express the biosensor HyPer in single isolated muscle fibres. Basically, our protocol consisted of the subcutaneous microinjection of the pHyPer-cyto vector between FDB muscle and pad skin followed by electroporation of the same muscle. After 5 days, we proceeded to isolate the muscle fibres from the FDB muscle. Once the fibres were cultured on plates, we assessed biosensor expression in the fibres by fluorescence microscopy. Fibres that expressed HyPer were located, and then live cell fluorescence microscopy experiments were conducted to assess the functionality of HyPer as a hydrogen peroxide biosensor.

**Figure 3:**
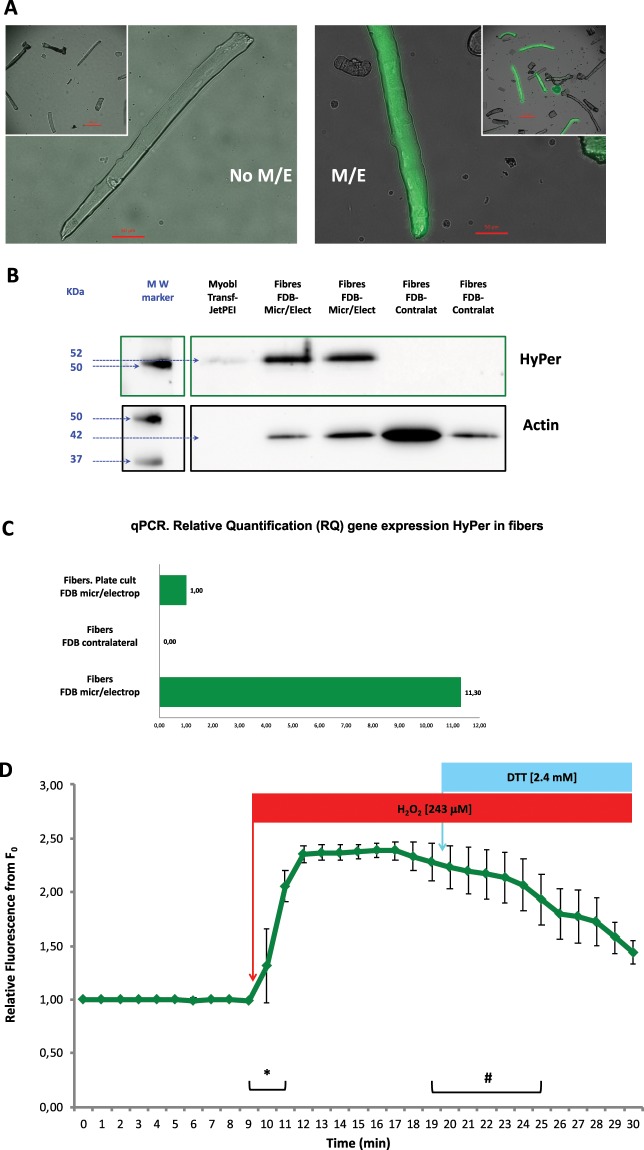
Single isolated muscle fibres expressing HyPer after microinjection and electroporation of the pHyPer-cyto vector in the FDB muscle in mice. (**A**) No M/E (left) presents a merged bright-field and fluorescence microscopy image of fibres isolated from the contralateral FDB (negative control) that had not undergone microinjection or electroporation. Inset, low magnification image of the same isolated fibre culture. Scale bars 50 μm and 200 μm (inset). M/E (right) presents a merged bright-field and fluorescence microscopy image of fibres from the FDB muscle that had been previously microinjected with pHyPer-cyto vector and electroporated five days before fibre isolation. (**B**) Cropped immunoblotting images of HyPer protein expression in isolated skeletal muscle fibres. Positive control: C2C12 myoblasts transfected with pHyPer-cyto vector using the transfection reagent JetPEI. Fibres from the FDB muscle that had previously been microinjected with the pHyPer-cyto vector and electroporated five days before fibre isolation. Fibres isolated from the contralateral FDB (negative control) that had not undergone microinjection or electroporation. The MW marker represents the lane where molecular weight protein reference markers were resolved in the immunoblot. HyPer and actin proteins were detected. Full-length immunoblot image in Supplementary Information. (**C**) Gene expression of the HyPer DNA coding sequence in isolated skeletal muscle fibres. Relative quantification (RQ) via qPCR of the HyPer mRNA transcript; the housekeeping gene used for normalization was the mouse beta-actin gene. The reference used for the relative quantification of gene expression were fibres isolated from the FDB muscle that had previously been microinjected with the pHyPer-cyto vector and electroporated five days before fibre isolation; these fibres were cultured on plates after fibre isolation. The negative controls were fibres isolated from the contralateral FDB muscle that had not undergone microinjection or electroporation. The third sample consisted of fibres isolated from the FDB muscle that had previously been microinjected with the pHyPer-cyto vector and electroporated five days before fibre isolation; the fibres from this sample were obtained directly after fibre isolation without fibre culture. (**D**) HyPer fluorescence emission from individual single skeletal muscle fibres isolated from the FDB muscle that had previously been microinjected with the pHyPer-cyto vector and electroporated five days before fibre isolation. Fluorescence was monitored every minute over a 30-minute time course. The mean values of relative fluorescence are presented ±s.e.m., n = 4 independent fibres. Single skeletal muscle fibres were exposed to H_2_O_2_ at a final concentration of 243 μM in medium at the 9–10 min time point and were exposed to DTT at a final concentration of 2.4 mM in medium at 19–20 min time point. * and ^**♯**^ statistically significant (p < 0.05) according to Student’s *t*-test.

Figure 3A shows isolated single muscle fibres from FDB muscle that had been previously microinjected with the pHyPer-cyto vector and electroporated five days before fibre isolation. A substantial number of viable fibres in the plate expressed the biosensor HyPer (Fig. 3A-M/E). Fibres isolated from the contralateral FDB muscle (negative control) from the same mouse did not emit fluorescence, indicating that there was no HyPer expression in these cells, as expected (Fig. 3A-No M/E).

Figure 3B presents an immunoblotting image from the analysis of HyPer expression in cultures of single muscle fibres isolated either from FDB muscle that had been previously microinjected and electroporated with pHyPer-cyto vector or the contralateral FDB muscle (negative control for pHyPer-cyto vector microinjection and electroporation). A positive control (C2C12 myoblasts transfected with pHyPer-cyto vector) for HyPer protein expression in muscle cells was included as reference. Fibres from microinjected and electroporated FDB reveal intense staining for HyPer. However, fibres from the contralateral FDB (negative control) clearly showed a lack of HyPer expression (Fig. 3B).

The analysis of gene expression by qPCR shows that the coding DNA sequence of HyPer was transcribed into mRNA in cultures of fibres isolated from the FDB muscle that had been previously microinjected with pHyPer-cyto vector and electroporated five days before fibre isolation. This was the reference (1.00) for the relative quantification of HyPer sequence expression in this analysis. As expected, the cultures of fibres isolated from the contralateral FDB showed the absence of transcribed mRNA corresponding to the HyPer coding sequence. Fibres isolated from FDB muscle that had been previously microinjected with pHyPer-cyto vector and electroporated ten days before fibre isolation but had not been cultured (stored in suspension at −80 °C) showed a relative expression of the HyPer coding sequence that was 11.30 times higher than that of the reference (1.00) for HyPer coding sequence expression (Fig. 3C).

The assessment of HyPer function as a specific biosensor for hydrogen peroxide in fibres that expressed HyPer, which had been isolated from FDB muscle microinjected with pHyPer-cyto vector and electroporated five days before fibre isolation, was undertaken with live cell fluorescence imaging experiments under the microscope. The results of these experiments are presented in Fig. 3D, which shows the fluorescence imaging analysis. Independent fibres from different cultures that were positive for HyPer expression were located under the live-cell microscope. Then, live cell fluorescence imaging experiments were carried out to record in real time the fluorescence emission (HyPer fluorescence) from fibres that expressed HyPer during a time course of 30 min by recording images every minute. The addition of hydrogen peroxide to the medium (final [H_2_O_2_] 243 μM) at the 9–10 min time point of the time course experiment evoked a significant intracellular fluorescence increase within 2 min in fibres that expressed HyPer. The addition of dithiothreitol (final [DTT] 2.4 mM) at the 19–20 min time point of the time-lapse experiment evoked a significant decrease in intracellular fluorescence within 6 min (Fig. 3D). This demonstrated and validated the premise that the biosensor HyPer that was expressed in single muscle fibres was functional and effective for the detection of changes in the intracellular concentration of hydrogen peroxide.

### Monitoring intracellular H_2_O_2_ in single isolated muscle fibres after extracellular H_2_O_2_ generation and consumption by enzymatic systems

**Figure 4:**
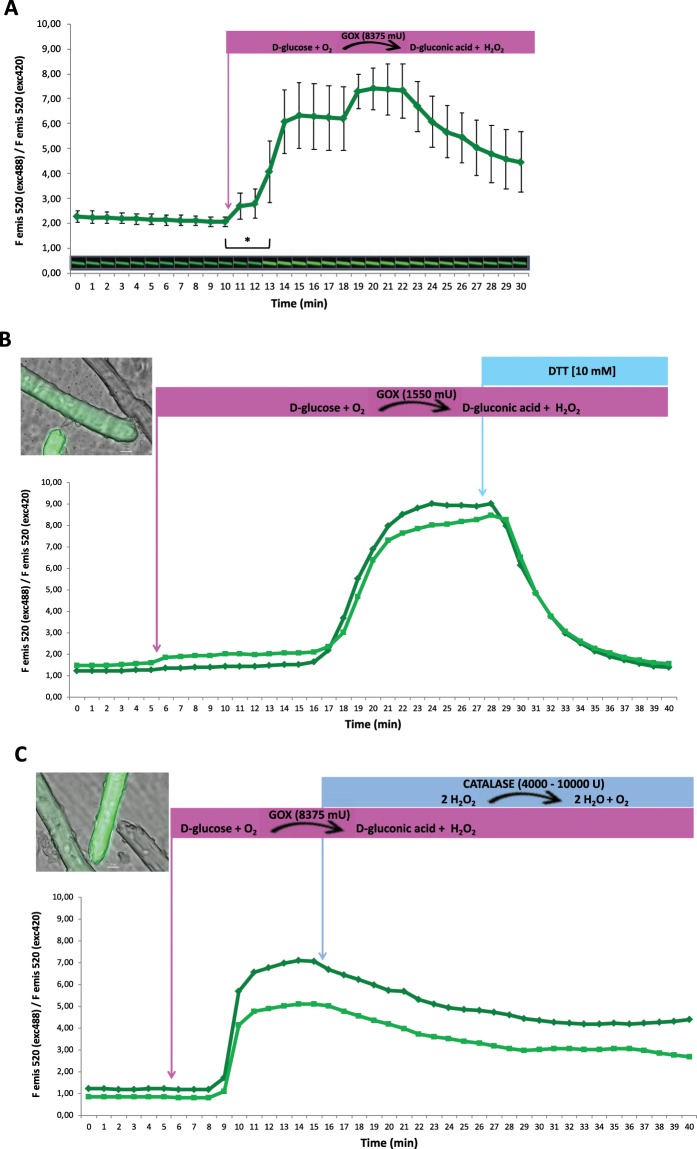
Monitoring of intracellular H_2_O_2_ in single isolated muscle fibres after extracellular H_2_O_2_ generation and consumption by enzymatic systems. (**A**) Fibres expressing the HyPer biosensor. The rate of fluorescence (the fluorescence emission at 520 nm after excitation at 488 nm divided by the fluorescence emission at 520 nm after excitation at 420 nm). The rate of HyPer fluorescence was recorded every minute over a 30-minute time course. Mean rate of HyPer fluorescence ± s.e.m., n = 5 independent fibres. Glucose oxidase (GOX) at 8375 mU was added to the medium after 10 min and was maintained in the medium during the following 20 min. Above the X axis, fluorescence images are presented (emission 520 nm, excitation 488 nm) from one representative fibre that were measured at every time point over the 30-minute time course. * statistically significant (p < 0.05, Student’s *t*-test) compared with the mean rate of HyPer fluorescence just before GOX incorporation into the medium at the 10 min time point. (**B**) Fibres that expressed HyPer2 are shown. The rate of fluorescence (fluorescence emission at 520 nm after excitation at 488 nm divided by the fluorescence emission at 520 nm after excitation at 420 nm) was recorded every minute during a 40-min time course. Glucose oxidase (GOX) at 1550 mU was added into the medium after 5 min and was maintained in the medium during the following 35 min. DTT was added into the medium after 27 min and was maintained for the rest of the time course (13 min) at a final concentration of 10 mM. The image shows three fibres: one fibre without HyPer2 expression and two fibres that expressed HyPer2 with different levels of expression; HyPer2 fluorescence was recorded and is presented on the time course graph. (**C**) Fibres that expressed HyPer2 are shown. The rate of fluorescence (fluorescence emission at 520 nm after excitation at 488 nm divided by the fluorescence emission at 520 nm after excitation at 420 nm) was recorded every minute during a 40-min time course. Glucose oxidase (GOX) at 8375 mU was added into the medium after 5 min and was maintained in the medium during the following 35 min. Catalase at 4000–10000 U was added into the medium after 15 min and was maintained for the rest of the time course (25 min). The image shows three fibres: one fibre did not express HyPer2, and the other two fibres expressed HyPer2 with different levels of expression. HyPer2 fluorescence was recorded in these two fibres, and the rate of fluorescence over time is presented on the graph.

Fibres expressing HyPer were monitored by measuring the rate of HyPer fluorescence (HyPer fluorescence emission at 520 nm after excitation at 488 nm divided by HyPer fluorescence emission at 520 nm after excitation at 420 nm) over a 30 min time course. Just after the 10 min time point, 8375 mU glucose oxidase (GOX) was added into the medium. GOX catalyses the transformation of D-glucose and O_2_ to D-gluconic acid and H_2_O_2_ in medium. H_2_O_2_ may diffuse through the sarcolemma into the fibre cytosol, where it can react with HyPer. A significant increase in HyPer fluorescence was observed 2 min after GOX addition, which was maintained over the rest of the time course experiment and reached a maximum 12 min after GOX addition (Fig. 4A).

Two fibres expressing HyPer2 with different levels of expression in the same culture preparation were monitored over a 40 min time course. A total of 1550 mU GOX was added to the medium after the 5 min time point, and an increase in the HyPer2 fluorescence rate was detected at the 18 min time point, reaching a maximum at approximately the 23–27 min time point. DTT (dithiothreitol; 10 mM) was added into the medium just after the 27 min time point. DTT evoked a dramatic decrease in the HyPer2 fluorescence rate in both fibres, which reached the basal value at the end of the time course (Fig. 4B).

Finally, two fibres that expressed HyPer2 with different intensities in the same culture were monitored for changes in the intracellular H_2_O_2_ concentration over a 40 min time course. GOX (8375 mU) was added to the medium after 5 min, which evoked a rapid increase in the HyPer2 fluorescence rate after 4 min of GOX incubation. This indicated that H_2_O_2_ generated in the medium by GOX diffused into the fibre cytosol and was detected by the HyPer2 biosensor. In addition, 4000–10000 U catalase was added to the medium just after the 15 min time point. Catalase reacts with and neutralizes H_2_O_2_, which is generated by GOX, to generate H_2_O. Thus, the H_2_O_2_ concentration in the medium was decreased. However, it was sufficient to diffuse into the fibres. This was reflected by the HyPer2 fluorescence rate, which was slightly and progressively reduced when catalase was present in the medium (Fig. 4C).

## Discussion

Belousov and collaborators developed the hydrogen peroxide biosensor HyPer in 2006^25^. Since then, HyPer has been used in different experimental cellular and organismal models to study the role of hydrogen peroxide as a signalling molecule in different cellular processes^2^. However, studies that report the use of HyPer in skeletal muscle have been limited and sporadic. In two of these studies, the coding sequence of HyPer that was inserted in an expression vector (pHyPer-Cyto-vector) was transfected into skeletal muscle fibres using a method based on the use of the lipid transfection reagent Lipofectamine^31,32^. However, there were no details regarding the efficiency of HyPer expression in those studies. The reported images of transfected fibres seemed to show that the viability of the fibres was compromised, and it was difficult to distinguish the typical transverse striations of isolated single skeletal muscle fibres^31,32^. Pearson and collaborators published a study in 2014 in which they used HyPer as a hydrogen peroxide biosensor in single isolated skeletal muscle fibres. They performed transduction techniques in which the coding sequence of HyPer was cloned into a recombinant adeno-associated viral vector (rAAV) to generate vector particles that were transduced into isolated skeletal muscle fibres^33^. In that study, the fibres transduced with viral particles appeared viable, and the transversal striations were obvious^33^. Apart from the previous three publications, we have not found any other studies in which HyPer was used in skeletal muscle cells.

To move ahead in redox biology research in skeletal muscle, it is necessary to use biosensors that detect ROS, RNS or redox states in skeletal muscle cells. One of these biosensors is HyPer, but there is a need for reliable experimental models that combine biosensors with typical cellular models for research in skeletal muscle, such as myoblasts, C2C12 myotubes, and the isolated single skeletal muscle fibre model. In addition, it would be desirable that those models and methods were available for conventional research laboratories so that this methodology may be more widely utilized.

In our study, we presented a gradual approach to establish reliable methods for using HyPer in three models used in skeletal muscle research: C2C12 myoblasts, C2C12 myotubes and single skeletal muscle fibres. We report the expression of HyPer biosensor in C2C12 myoblasts using chemical transfection with a polymeric reagent, JetPEI, which facilitated the internalization of the pHyPer-cyto vector into the cytoplasm of C2C12 myoblasts. Then, the cellular transcription and translation machinery resulted in the expression of the HyPer biosensor in the cytoplasm of these cells. The efficiency of transfection in our study was low. However, HyPer gene expression was detected at the transcriptional level (mRNA) and protein translation. Although the number of cells expressing HyPer was low, it was sufficient for carrying out the functional analysis of this biosensor using quantitative fluorescence microscopy image analysis, since the methodological approach we used made it possible to carry out the analysis on a single cell or even in a region of a cell to detect and quantify the fluorescence emitted in real time. The functionality of HyPer as a specific biosensor for H_2_O_2_ detection was assessed in C2C12 myoblasts. When H_2_O_2_ was added to the medium, it crossed the plasma membrane of the myoblast, and once it was in the cytoplasm, it reacted with the protonated form of HyPer, resulting in HyPer oxidation and subsequent fluorescence emission from oxidized HyPer^34^. It is well known in the field that H_2_O_2_ crosses the plasma membrane by passive diffusion, and some studies have reported that specific transporters, such as aquaporins, mediate the flux of H_2_O_2_ through the plasma membrane^35^. A 476 μM H_2_O_2_ concentration in the medium might represent 50-5 μM H_2_O_2_ intracellular concentration^21,36,37^. This is at the limit of the physiological range, and it could even be pathological^37^, since cells showed signs of toxicity 1 hour after exposure to H_2_O_2_. To assess the reversibility of the HyPer biosensor, C2C12 myoblasts were exposed to a potent reductant, dithiothreitol (DTT), following H_2_O_2_ exposure in the same experiment. DTT evoked a dramatic and significant decrease in fluorescence emitted by HyPer. This may be explained by the strong reducing power of DTT. Which evoke the chemical reduction of oxidized HyPer and transform it into reduced or protonated HyPer, that emits a decreased fluorescence level^34^. Therefore, it was clearly demonstrated that the expression of HyPer in C2C12 myoblasts acted as a biosensor that detected the increase in intracellular H_2_O_2_ generation and changes in the intracellular redox state.

C2C12 myotubes are derived from the fusion of C2C12 myoblasts that adopt a multinuclear tubular cell shape^28^. These cells are difficult to be transfected with DNA vectors. In the first stage, we tried the transfection in C2C12 myotubes with pHyPer-cyto vector and JetPEI, and the results were limited. Then, we decided to adopt a new approach and used another transfection protocol based on the chemical polymeric transfection reagent Viromer RED (Lypocalyx). Viromer RED provided improved results compared with the JetPEI, and we found proper myotubes expressing HyPer. In addition, HyPer gene expression was higher in C2C12 myotubes that had been transfected with Viromer RED compared with those transfected with JetPEI. Thus, it appears that the Viromer RED transfection protocol is an appropriate approach to facilitate the expression of HyPer in C2C12 myotubes, and it is plausible that this protocol might be suitable for the transfection and expression of other DNA vectors in C2C12 myotubes. The functionality of the HyPer as a reversible biosensor that detects changes in the intracellular concentration of H_2_O_2_ was evidenced when myotubes were exposed to H_2_O_2,_ and the fluorescence of HyPer increased rapidly, indicating that H_2_O_2_ had diffused through the plasma membrane and reacted and oxidised HyPer. This led to an increase in the fluorescence emission of HyPer. In contrast, when DTT was added to the medium, HyPer fluorescence decreased.

The main objective of this study was to develop a plausible approach that allows the expression of the H_2_O_2_ biosensor HyPer in single isolated skeletal muscle fibres. As discussed above, we developed strategies to achieve the expression of the biosensor HyPer in two *in vitro* models, C2C12 myoblasts and myotubes. However, these models are limited in the study of important skeletal muscle disorders, such as insulin resistance in diabetes and ageing. Single skeletal muscle fibres are isolated from the *flexor digitorum brevis* muscle in the mouse foot. This is a great advantage, since this model recapitulates the insulin resistance observed in type 2 diabetes in skeletal muscle, and it is possible to study mouse strains with accelerated ageing or normal ageing at different ontogenetic stages. Different studies in recent years have suggested a potential role for H_2_O_2_ in insulin resistance in skeletal muscle in type 2 diabetes^13,38,39^. However, the mechanisms by which H_2_O_2_ is involved in the control or modulation of these processes are only partially known. The approach presented in this study provides a plausible methodology to induce the expression of the H_2_O_2_ biosensor HyPer in skeletal muscle fibres. This is a new research avenue that will facilitate investigation to increase the understanding of the role of H_2_O_2_ in insulin resistance in skeletal muscle in diabetes and ageing.

In the first stage, we tried to expand the approaches used for the expression of HyPer in C2C12 myoblasts and myotubes to skeletal muscle fibres to produce HyPer expression in these cells. Despite the fact that we performed different modifications of the methodology of transfection, the results were unsatisfactory, and HyPer expression was not present in the fibres. Then, we used a different approach based on the direct microinjection of the pHyPer-cyto vector into the *flexor digitorum brevis* muscle followed by direct electroporation of this muscle^30^. We incorporated some adaptations and achieved the expression of the H_2_O_2_ biosensor HyPer in fibres from the FDB muscle. Following fibre isolation^21,40^, we obtained single isolated fibres that expressed HyPer. Fibres in cultures expressing HyPer are an appropriate model to conduct fluorescence microscopy experiments for the analysis of the intracellular presence, flux and generation of H_2_O_2_. In addition, the expression of HyPer in fibres was assessed by immunoblotting and gene expression analysis. The biosensor HyPer was capable of detecting or sensing intracellular H_2_O_2_ in fibres, 2.5 μM according to recent estimations^37^. The reversibility of HyPer was demonstrated when DTT was added to the medium, and this produced a significant decrease in HyPer fluorescence, that may have been due to the chemical reduction by DTT of HyPer. Therefore, HyPer expressed in single muscle fibres was a functional and effective biosensor to detect changes in the intracellular concentration and flux of hydrogen peroxide in muscle fibres.

The detection and monitoring of physiological intracellular changes in H_2_O_2_ in real time is a challenging endeavour in the field of redox biology. In this study, we developed new and improved approaches that use the HyPer biosensor to analyse the flux and diffusion of H_2_O_2_ into the cytoplasm of isolated skeletal muscle fibres. Thus, skeletal muscle fibres in culture were exposed to glucose oxidase (GOX). This is considered an enzymatic method to generate H_2_O_2_^38,41^. H_2_O_2_ may cross the sarcolemma and diffuse into the cytosol of fibres, as shown by our experiments, in which fibres that expressed the HyPer biosensor showed an increase in the HyPer fluorescence rate after GOX initiated the reaction to generate H_2_O_2_, indicating that H_2_O_2_ diffused into fibres and oxidized HyPer and thereby increased fluorescence emission until the fluorescence started to decay, which was probably due to the protonation (reduction) of HyPer by reducing enzymes such as glutaredoxins^34^ and a decreased intracellular concentration of H_2_O_2_, since intracellular catalase and glutathione peroxidase reactions might neutralize H_2_O_2_ into H_2_O^12^. Therefore, HyPer is able to sense intracellular H_2_O_2_ fluctuations produced by H_2_O_2_ generated by the extracellular enzymatic system GOX.

We examined another version of HyPer, the HyPer2 biosensor, which had been reported to have a higher dynamic fluorescence range and likely a higher sensitivity for the detection of H_2_O_2_^27,34^. The aim was to detect the minimal intracellular changes in H_2_O_2_ that might play an important role in redox signalling^37^. Fibres expressing HyPer2 were analysed, and GOX was added to the medium at a proportion five times lower than that used in the previous experiments. The HyPer2 fluorescence rate started to increase after GOX addition with a higher dynamic range compared to that of HyPer, despite the difference of GOX activity. The HyPer2 biosensor appears to be more sensitive and to produce higher fluorescence signals than HyPer. Therefore, the HyPer2 biosensor is more appropriate than HyPer for detecting intracellular fluctuations of H_2_O_2_ in muscle fibres.

Finally, to mimic in some way the physiology of the cell, we examined whether the HyPer2 biosensor had enough sensitivity to detect changes in the intracellular H_2_O_2_ concentration when an antioxidant system (catalase) was operating together with a H_2_O_2_ generation system (GOX). Muscle fibres displayed a clear increase in the HyPer2 fluorescence rate after GOX was added to the fibre medium. This indicated that the H_2_O_2_ fluxed into the cytosol of the fibre. However, when catalase was added to the fibre medium, H_2_O_2_ was neutralized to H_2_O. Thus, a proportion of the H_2_O_2_ generated by GOX was quenched, and as a consequence, the extracellular H_2_O_2_ concentration was diminished and H_2_O_2_ flux to the cytosol of the fibre was reduced. This was detected intracellularly and monitored by the slow and progressive decrease in the HyPer2 fluorescence rate, which started just after catalase was added to fibre medium and the neutralization of H_2_O_2_ into H_2_O commenced. Therefore, the intracellular fluctuations of H_2_O_2_ were measured and monitored according to the HyPer2 fluorescence rate in real time in an experimental system, GOX/catalase, that might mimic the pathophysiological conditions of skeletal muscle.

In conclusion, the HyPer biosensor is a powerful tool to detect and measure the intracellular concentration of H_2_O_2_ in real time in models of skeletal muscle, such as C2C12 myoblasts, C2C12 myotubes and single skeletal muscle fibres. The HyPer2 biosensor might be more appropriate than HyPer for measuring low H_2_O_2_ signals, since HyPer2 seems to be more sensitive and to have a higher fluorescence dynamic range. The outcome of this study describes the basics of the use of biosensors to investigate H_2_O_2_ as a signalling molecule in skeletal muscle cells. This will be crucial for increasing the understanding of the role of H_2_O_2_ in the pathophysiology of skeletal muscle. Furthermore, the methodological approaches described in this study are potentially applicable to assaying other new biosensors that are already available for specific RONS, signalling molecules or second messengers, and many other biosensors will hopefully be developed in the near future to investigate pathophysiological processes in the field of redox biology.

## Methods

### Reagents

Hydrogen peroxide solution (30 wt% in water), dithiothreitol (DTT), glucose oxidase (GOX) and catalase (CAT) were obtained from Sigma-Aldrich.

### Skeletal muscle cell culture

C2C12 myoblasts are a mouse skeletal muscle cell line (CRL-1772, American Type Culture Collection). C2C12 myoblasts were cultured in growth medium, which consisted of Dulbecco’s modified Eagle medium (DMEM, Sigma-Aldrich) with 10% (v/v) foetal bovine serum (FBS, Invitrogen) supplemented with 2 mM L-glutamine (Sigma-Aldrich), 50 i.u. penicillin and 50 μg ml^−1^ streptomycin (Sigma-Aldrich). C2C12 growth and expansion were carried out in an incubator with 5% CO2 in humidified air at 37 °C. C2C12 myotubes were obtained from myoblasts that had been previously induced to differentiate. Differentiation involved growing myoblasts to 80–90% confluency and then replacing the growth medium with differentiation medium (DMEM with 2% (v/v) horse serum (Invitrogen) supplemented with 2 mM L-glutamine, 50 i.u. penicillin and 50 μg ml^−1^ streptomycin). Cellular differentiation was maintained for 7 days, and during this time, mononuclear myoblasts fused and transformed into multinuclear myotubes with a tubular morphology.

### Animals

Three female and 2 male 3-month-old C57BL/6J mice were used in this study. Procedures involving animals were approved by the Bioethics Committee of the University of Salamanca in accordance with the Spanish (RD 53/2013) and European Union (2010/63/UE) guidelines for animal experimentation.

### Isolated skeletal muscle fibres

Single skeletal muscle fibres were isolated from the *flexor digitorum brevis* (FDB) muscle in mice according to the protocol established by Palomero and collaborators^21,40^. Once the skeletal muscle fibres were isolated, they were maintained in a 5% CO_2_ humidified atmosphere at 37 °C for 24–48 h in an incubator until the day the fluorescence microscopy experiments were conducted.

### pHyPer-cyto vector transfection in myoblasts and myotubes

Transfection of the pHyPer-cyto vector in C2C12 myoblasts was performed using a transfection technique based on the use of the cationic polymer transfection reagent JetPEI (JetPEI, Polyplus transfection). According to the manufacturer’s instructions, the pHyPer-cyto vector and JetPEI were diluted separately in 150 mM NaCl, and equal volumes of each solution were mixed to obtain the transfection complexes, which were created at a ratio of 1 μg DNA pHyPer-cyto vector: 2 μl JetPEI. These complexes were added to C2C12 myoblasts in culture at 70–80% confluency and maintained in culture for 24–72 h. The transfection efficiency was monitored by fluorescence microscopy every 12 h until the functional fluorescence microscopy experiments were conducted or the myoblasts were collected for immunoblotting or qPCR analysis.

Transfection of the pHyPer-cyto vector into the C2C12 myotubes was performed using a transfection technique based on the use of a polymeric transfection reagent, Viromer RED (Viromer RED, Lypocalyx). C2C12 myoblasts were cultured until they reached 70–80% confluency, and then transfection was performed at a ratio of 1.24 μg DNA (pHyPer-cyto vector): 1 μl Viromer RED according to the manufacturer’s instructions with optimization in our laboratory. Twenty-four hours later, the growth medium was switched to differentiation medium, and the cells were maintained in differentiation medium for 6 days. Over that time, the myoblasts fused and differentiated into myotubes. The transfection efficiency and HyPer expression in myoblasts/myotubes were monitored by fluorescence microscopy every 24 h until the functional fluorescence microscopy experiments were carried out or myotubes were collected for qPCR analysis. In addition, a modification of the transfection protocol was performed; the C2C12 myoblasts were transfected at a ratio of 2.48 μg DNA pHyPer-cyto vector: 1 μl Viromer RED, and 72 h later, the transfected cells were collected for immunoblotting and qPCR analysis.

### Transfection of pHyPer-cyto vector or pC1-HyPer-2 into FDB muscle from mice *in vivo*

Microinjection of either the pHyPer-cyto or pC1-HyPer-2 vector was performed in the FDB muscle, and the contralateral FDB was considered a control (no microinjection) according to the protocol established by DiFranco and collaborators^30,42^ with modifications adapted in our laboratory. Mice were maintained under gas anaesthesia (3% isoflurane) during the microinjection procedure. Microinjection was performed using a micro syringe provided with a 0.3 mm (30G) × 8 mm needle (BD Micro-Fine+). First, 10 μl sodium chloride hyaluronidase solution (2 mg/ml) (SIGMA) was injected subcutaneously into the mouse pad between the FDB muscle and the skin. After 60 min of stabilization, 10 μl of pHyPer-cyto (5 μg/μl) or pC1-HyPer-2 (3 μg/μl) vector was injected into the same FDB muscle. After 15 min of stabilization, two stainless steel needle electrodes (0.3 mm (30G) × 8 mm) were placed subcutaneously and perpendicular to the FDB longitudinal axes on the proximity of the injection area, with a 0.5-cm separation between the electrodes. Then, electroporation was applied using an electrical stimulator (stimulator CS type 223, Hugo Sachs Elektronik, Harvard Apparatus). Electroporation consisted of the application of 20 square positive pulses, with 5 s intervals between pulses, a 50 V pulse potential and a 10 ms pulse amplitude. After electroporation, the mice were allowed to recover from anaesthesia and maintained for five days until muscle fibre isolation.

### Genetically encoded HyPer and HyPer2 sequences

The HyPer DNA-encoding sequence was inserted into the pHyPer-cyto vector (Evrogen, FP-941). The pHyPer-cyto vector was used for C2C12 myoblast and myotube transfection to induce the expression of the HyPer biosensor in these cells. The pHyPer cyto vector was microinjected into the FDB muscle followed by electroporation in that muscle, which produced the expression of the HyPer biosensor in muscle fibres. The pHyPer-cyto vector had been previously amplified in DH5alfa *E. coli* followed by plasmid purification using a Plasmid Midiprep Kit (GeneJET, Thermo Scientific) to obtain the amount of DNA required for transfection and microinjection.

The HyPer2 DNA-encoding sequence was inserted into the plasmid pC1-HyPer-2. This was a gift from Vsevolod Belousov (Addgene plasmid repository, plasmid 42211). HyPer2 is a genetically encoded sensor for H_2_O_2_ with an expanded dynamic range compared with that of the HyPer biosensor^27^. pC1-HyPer-2 was multiplied and purified following the same procedure as that used for the pHyPer-cyto vector.

### Immunoblotting

Protein was extracted from C2C12 myoblasts or C2C12 myotubes that had been previously transfected with JetPEI or Viromer RED and pHyPer-cyto vector. Similarly, protein was extracted from mature skeletal muscle fibres isolated from a FDB muscle that had been previously microinjected with the pHyPer-cyto vector and subjected to electroporation. These cell samples were homogenized in homogenization buffer containing 10 mM TRIS–HCl pH 7.4, 100 mM NaCl, 1 mM EDTA, 1 mM EGTA, 1% (v/v) Triton X-100, 10% (v/v) glycerol, 0.1% (w/v) sodium dodecyl sulphate, 0.5% (w/v) deoxycholate, and protease inhibitor cocktail (Sigma-Aldrich). The homogenates were centrifuged, and the supernatants were collected to obtain the protein samples to be resolved by electrophoresis. The total protein content was determined using the bicinchoninic acid kit for protein determination (Sigma-Aldrich), and then the samples were stored at −80 °C until electrophoresis was performed under denaturing conditions. A total of 30 μg of each protein sample was mixed with an equal volume of loading buffer (2X Laemmli Buffer), and this mixture was incubated at 95 °C for 10 min to obtain the denatured protein samples, which were loaded on an SDS-PAGE gel (4–10% polyacrylamide). In addition, a molecular weight protein marker (Bio-Rad) was loaded in a lane of the same gel. Electrophoresis was performed (200 V for 45 min), and the resolved proteins were transferred to a nitrocellulose membrane by applying an electrical current of 100 V and 350 mA for 60 min. Nonspecific binding sites in the membranes were blocked by incubation for one hour with 5% (w/v) powdered non-fat milk. The membranes were incubated overnight at 4 °C with the primary antibody in TBS-T with 1% bovine serum albumin (BSA). The primary antibodies were anti-HyPer (Anti-Tag (CGY)FP antibody, Evrogen, AB121) (1:1000 dilution for myoblasts and 1:2000 dilution for myotubes and fibres) and anti-actin (Sigma, A2103) (1:5000 for myoblasts and 1:2500 for fibres). The secondary antibody was an ECL donkey anti-rabbit IgG (Amersham, NA934VS) (1:10000 dilution). The membranes were incubated with a chemiluminescence reagent (Clarity Western ECL Blotting Substrate, Bio-Rad), and the image signals were visualized by chemiluminescence using a digital recording CCD imaging system (MicroChemi, Bio-Imaging Systems). Digital images were obtained for further analysis and presentation in figures.

### Quantitative real-time PCR (qPCR)

RNA isolation and quantitative real-time PCR were performed as a core service of the University of Salamanca, Bancoadn (DNA National Bank Carlos III). RNA was extracted from C2C12 myoblasts, C2C12 myotubes or mature skeletal muscle fibres isolated from the FDB muscle according to a protocol based on the use of organic solvents and an easy-BLUE Total RNA Extraction Kit (Intron Biotechnologies). Total RNA was treated with DNAase I (Sigma-Aldrich) to digest any residual genomic DNA. RNA quality and purity were assessed by agarose gel electrophoresis. cDNA was synthesized from RNA according to the protocol for the High-Capacity cDNA Reverse Transcription kit (Applied Biosystems). qPCR was performed using the QuantStudio 7 Flex Real-Time PCR System, Fast 96-well plate and SYBR Green Reagent. The mRNA transcript levels were normalized to those of mouse beta-actin (mActb). The following primers were used: HyPer forward: 5′-GATTCCCACAGTTGGACCG-3′; HyPer reverse: 5′-TCTCGTTGGGGTCTTTGCTC-3′; mActb forward: 5′-CGAGGCCCCCCTGAAC-3′; mActb reverse: 5′-GCCTGGATGGCTACGTACATG-3′.

### Fluorescence microscopy and image analysis

C2C12 myoblasts, myotubes and single skeletal muscle fibres that expressed HyPer were placed under a fluorescence microscope (Live Cell Observer, Carl Zeiss) equipped with a chamber to maintain the temperature at 37 °C to conduct experiments to assess the functionality of HyPer in real time in these cells. Before starting the experiments in the microscope, the culture medium was removed from the cells and replaced by Krebs solution (119 mM NaCl, 2.5 mM KCl, 2.5 mM CaCl_2_-2H_2_O, 1.5 mM MgSO_4_-7H_2_O, 1.25 mM NaH_2_PO_4_, 26.2 mM NaHCO_3_, and 11.1 mM glucose, pH 7.4). The cells were maintained in Krebs solution at 37 °C for 30 min of image recording, and images were obtained every 1 min during the time-lapse with a 40× magnification objective (Objective LD Plan-Neofluar 40×/0.6 Corr Ph2 DIC, Carl Zeiss). The source of the excitation light was a light-emitting diode (LED) that generated 470 nm monochromatic light. Fluorescence images were obtained through a fluorescence cube with a 450/40 nm excitation filter, 495 nm beam splitter and 525/50 nm emission filter. Fluorescence images were acquired with a computer-controlled CCD camera (AxioCam MRm, Carl Zeiss) coupled to the microscope. To avoid light-induced damage to the cells, fluorescence images were acquired with the minimum exposure time that provided images with enough quality for quantification. All of the time-lapse images were acquired with the same exposure time, and this was maintained for every time-lapse experiment performed under the same conditions. The fluorescence image analysis was undertaken using the software (ZEN 2 blue edition, Carl Zeiss) included with the microscopy equipment. The fluorescence images recorded in every time-lapse experiment were analysed. Thus, a region of interest (ROI) was selected in a cell or fibre that expressed HyPer. In addition, another ROI was selected outside the cell or fibre to represent the background. The software provided the measurement, in terms of grey activated pixels, of the fluorescence intensity of the two ROIs. The net fluorescence of the cell or fibre expressing HyPer was obtained by subtracting the fluorescence value of the background ROI from the fluorescence value of the cell or fibre ROI, and this represented the raw net fluorescence of the cell or fibre expressing HyPer, which was determined at each time point in which an image was acquired during the time lapse experiment. The raw net fluorescence was transformed into the relative net fluorescence, which is more appropriate for statistical analysis, since this avoids differences in fluorescence intensity due to a potential difference in the expression of HyPer in cells. The relative net fluorescence was calculated for each time point of the time-lapse experiment by using the net raw fluorescence at the initiation of time-lapse experiment (rawF0) as the reference for normalization, which had a value of 1.00. The normalized fluorescence for the time point n (normFn) was calculated by dividing raw fluorescence at that time point (rawFn) by the rawF0. Thus, normFn = rawFn/rawF0.

The fluorescence image analysis of fibres that expressed the HyPer2 biosensor consisted of a ratiometric analysis. Thus, fluorescence images were obtained from two channels, green and blue. The image from the green channel indicated the fluorescence emission at 520 nm under excitation with 470 nm monochromatic LED using a set of filters (450/40 nm excitation filter, 495 nm beam splitter and 525/50 nm emission filter). The image from the blue channel indicated the fluorescence emission at 520 nm under excitation with UV light (at a range of approximately 420 nm) using a set of filters (395–440 nm excitation filter, 460 nm beam splitter and 470 LP nm emission filter). The imaging analysis was used to determine the ratiometric HyPer2 fluorescence in the fibres. Thus, the ratio = F emis 520 (exc488)/F emis 520 (exc420), where F emis 520 (exc488) is the net fluorescence from the green channel (ROI raw fluorescence minus the background raw fluorescence) and F emis 520 (exc420) is the net fluorescence from the blue channel (ROI raw fluorescence minus the background raw fluorescence).

### Statistical analysis

The values of the net relative fluorescence or rate of fluorescence are presented as the mean ± standard error of the mean (s.e.m.). Student’s *t*-test was used to detect the significant differences in the relative net fluorescence or the rate of fluorescence between two specific time points. Statistical significance was indicated at p < 0.05.

## Supplementary information

Full-length immunoblot images.
