# Supplementary material for: Expression and functional analysis of the hydrogen peroxide biosensors HyPer and HyPer2 in C2C12 myoblasts/myotubes and single skeletal muscle fibres
Source: Sci Rep. 2020 Jan 21;10:871. doi: 10.1038/s41598-020-57821-1 (PMC6972731; doi:10.1038/s41598-020-57821-1)
Supplement: Supplementary file 1 — Full-length immunoblot images. [file 41598_2020_57821_MOESM1_ESM.pdf]

Expression and functional analysis of the hydrogen peroxide biosensors HyPer and HyPer2 in C2C12 myoblasts/myotubes and single skeletal muscle fibres

Escarlata Fernández-Puente[1,4,5], Manuel A Sánchez-Martín[2,3,5], Jorge de Andrés[1], Lorena Rodríguez-Izquierdo[1], Lucía Méndez[3], Jesús Palomero*[1,4,5]

[1] *Department of Physiology and Pharmacology. University of Salamanca. Salamanca. Spain.*

[2] *Department of Medicine. University of Salamanca. Salamanca. Spain.*

[3] *Transgenic Facility Unit. University of Salamanca. Salamanca. Spain.*

[4] *Institute of Neurosciences of Castilla y León (INCyL). Salamanca. Spain.*

[5] *Institute of Biomedical Research of Salamanca (IBSAL), Salamanca, Spain*

** Corresponding author:*

Jesús Palomero (jespala@usal.es)

Department of Physiology and Pharmacology

Faculty of Medicine.

University of Salamanca.

Campus Miguel de Unamuno.

Av. Alfonso X El Sabio, s/n.

37007 Salamanca. Spain

FIGURE 1B Supplementary Information.

Full-length immunoblot image of Figure 1B

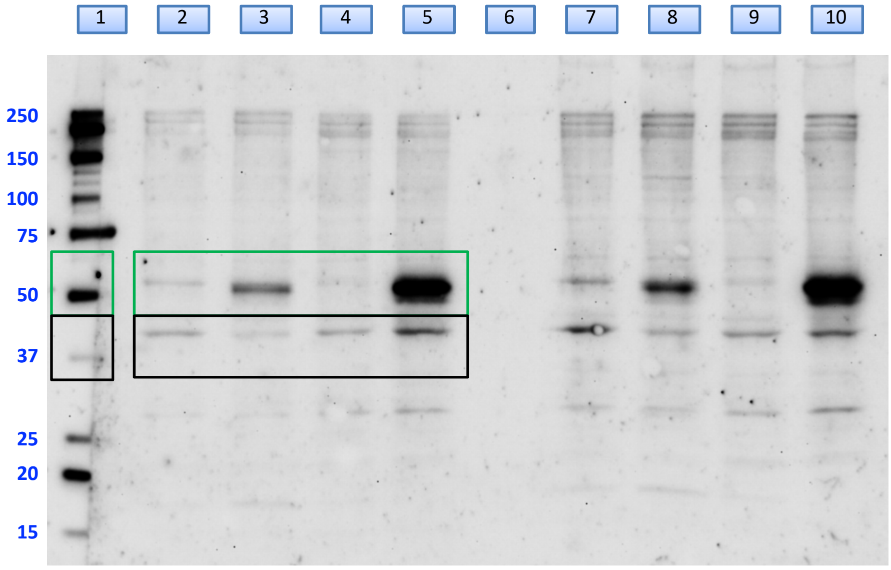

Full-length immunoblot image of Figure 1B. Cropped areas are depicted in green and black rectangles, which are displayed in Figure 1B. Molecular weight protein reference markers run in line 1. Samples run in lines 2, 3, 4 and 5. This Immunoblot was firstly probed with antibody against HyPer (green) and then with antibody against actin (black) and incubated concomitant with the secondary antibody.

FIGURE 2B Supplementary Information.

Full-length immunoblot image of Figure 2B

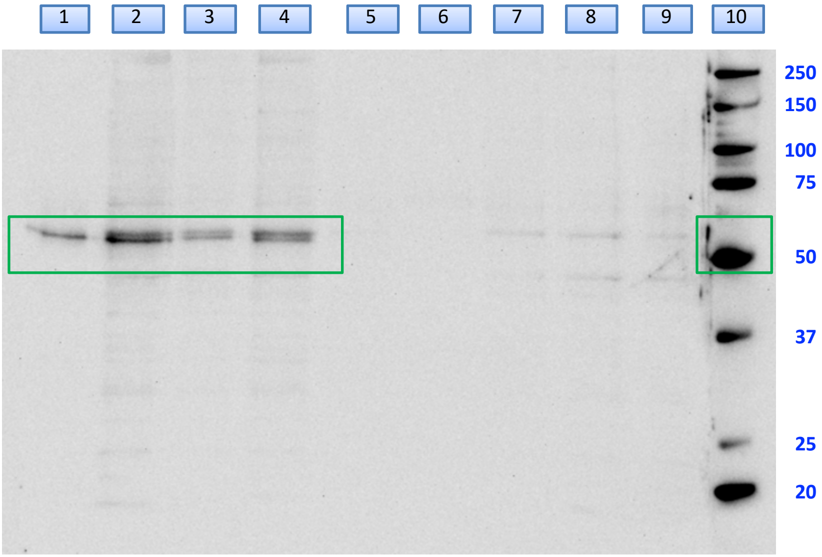

Full-length immunoblot image of Figure 2B. Cropped areas are depicted in green rectangles, which are displayed in Figure 2B. Molecular weight protein reference markers run in line 10. Samples run in lines 1, 2, 3 and 4. Cropped area of 50 KDa protein weight marker is placed on the left side in Figure 2B in order to be consistent with the format of other figures. Cropped image of samples in Figure 2B was obtained with higher exposure time to increase image contrast in order to improve visualization of bands.

FIGURE 3B Supplementary Information.

Full-length immunoblot image of Figure 3B

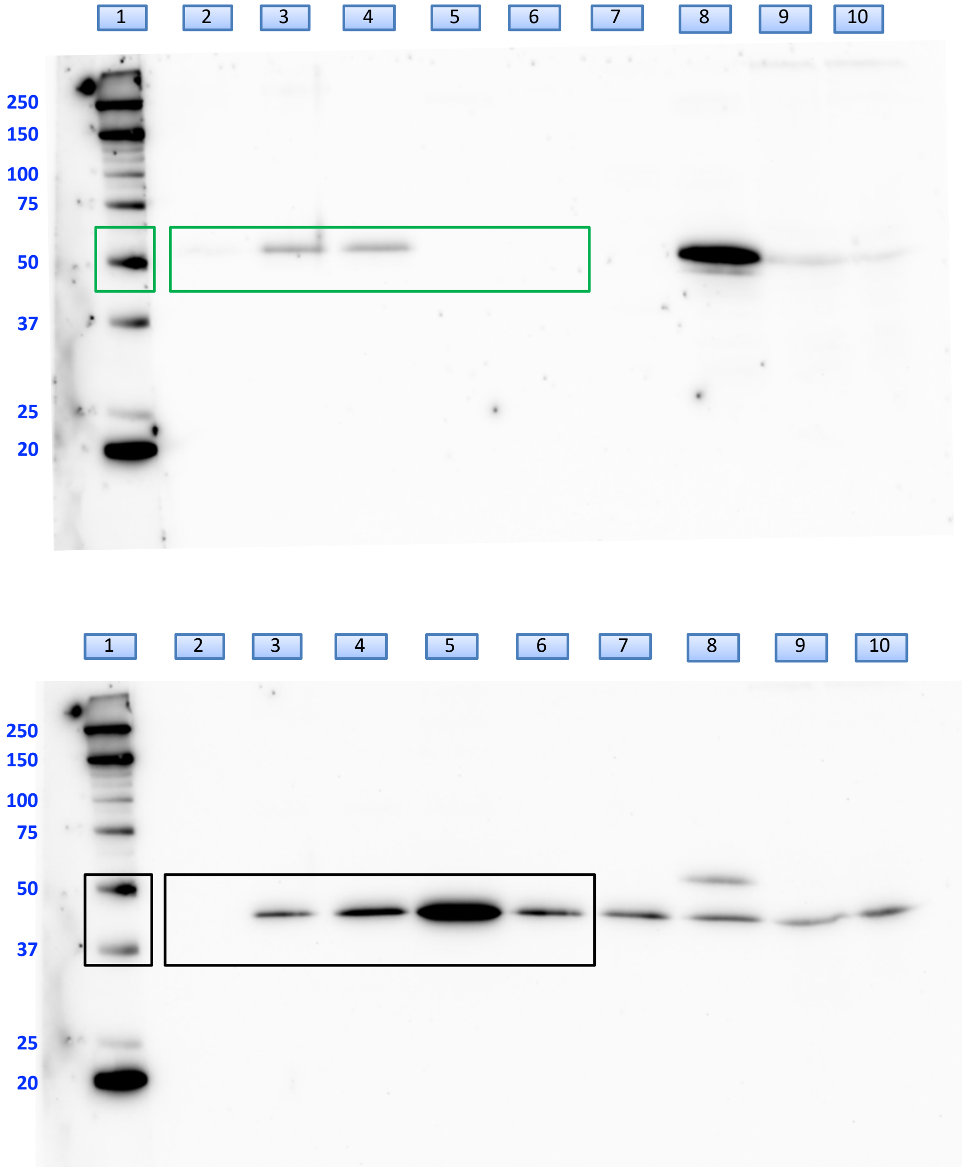

Full-length immunoblot images of Figure 3B. Cropped areas are depicted in green and black rectangles, which are displayed in Figure 3B. Molecular weight protein reference markers run in line 1. Samples run in lines 2, 3, 4, 5 and 6. Samples were probed with antibody against HyPer (upper immunoblot image) and with antibody against actin (lower immunoblot image). Cropped images of samples in Figure 3B were obtained with higher exposure time to increase image contrast in order to improve visualization of bands.
